# AntiFold: improved structure-based antibody design using inverse folding

**DOI:** 10.1093/bioadv/vbae202

**Published:** 2025-03-21

**Authors:** Magnus Haraldson Høie, Alissa M Hummer, Tobias H Olsen, Broncio Aguilar-Sanjuan, Morten Nielsen, Charlotte M Deane

**Affiliations:** Section for Bioinformatics, Department of Health Technology, Technical University of Denmark, Lyngby DK-2800, Denmark; Department of Statistics, University of Oxford, Oxford OX1 3LB, United Kingdom; Department of Statistics, University of Oxford, Oxford OX1 3LB, United Kingdom; Department of Statistics, University of Oxford, Oxford OX1 3LB, United Kingdom; Section for Bioinformatics, Department of Health Technology, Technical University of Denmark, Lyngby DK-2800, Denmark; Department of Statistics, University of Oxford, Oxford OX1 3LB, United Kingdom

## Abstract

**Summary:**

The design and optimization of antibodies requires an intricate balance across multiple properties. Protein inverse folding models, capable of generating diverse sequences folding into the same structure, are promising tools for maintaining structural integrity during antibody design. Here, we present AntiFold, an antibody-specific inverse folding model, fine-tuned from ESM-IF1 on solved and predicted antibody structures. AntiFold outperforms existing inverse folding tools on sequence recovery across complementarity-determining regions, with designed sequences showing high structural similarity to their solved counterpart. It additionally achieves stronger correlations when predicting antibody-antigen binding affinity in a zero-shot manner. AntiFold assigns low probabilities to mutations that disrupt antigen binding, synergizing with protein language model residue probabilities, and demonstrates promise for guiding antibody optimization while retaining structure-related properties.

**Availability and implementation:**

AntiFold is freely available under the BSD 3-Clause as a web server (https://opig.stats.ox.ac.uk/webapps/antifold/) and pip-installable package (https://github.com/oxpig/AntiFold).

## 1 Introduction

Antibodies are one of the largest classes of therapeutics, used to treat diseases including cancers, autoimmune conditions, and viral infections (Lu *et al.* 2020). Therapeutic antibody design is complex, requiring the optimization of numerous properties related to efficacy, manufacturability, and safety (Rabia *et al.* 2018).

Machine learning-based methods have shown promise in accelerating multiple steps in the antibody development pipeline (Hummer *et al.* 2022) by reducing liabilities such as immunogenicity (Marks *et al.* 2021, Prihoda *et al.* 2022, Tennenhouse *et al.* 2024) and aggregation (Makowski *et al.* 2023) or rationally optimizing for desirable properties such as binding affinity and developability (Makowski *et al.* 2022).

A guiding consideration in antibody optimization is selecting mutations that maintain the structure and therefore structure-mediated properties, ranging from stability to antigen binding mode. Protein inverse folding models are trained to predict sequence given structure (Li *et al.* 2014, O’Connell *et al.* 2018, Ingraham *et al.* 2019, Strokach *et al.* 2020, Jing *et al.* 2021,  Anand *et al.* 2022, Hsu *et al.* 2022, Dauparas *et al.* 2022) and can therefore be used to design novel sequences without altering the antibody backbone structure. Backbone-constrained design could enable the optimization of individual properties without disrupting others.

Antibodies demonstrate distinct structure and sequence properties as compared to general proteins (Stanfield and Wilson 2014, Regep *et al.* 2017) (Supplementary Fig. S1). While the framework (FR) regions are germline-encoded and relatively conserved, the complementarity-determining region (CDR) loops are hypervariable and form most of the antigen-binding contacts. Over two-thirds of CDRH3 loops have distinct structures not found in other general protein structures (Regep *et al.* 2017).

Multiple tools for antibody inverse folding design have recently been released, including AbMPNN (Dreyer *et al.* 2023) and IgMPNN (Shanehsazzadeh *et al.* 2023), based on the ProteinMPNN architecture (Dauparas *et al.* 2022). Furthermore, recent work has shown the promise of protein language and inverse folding models for guiding antibody affinity maturation through the identification of high-fitness, structurally constrained regions of the mutational landscape (Hie *et al.* 2023, Shanker *et al.* 2024, Outeiral and Deane 2024). However, the sequence recovery of existing tools for the CDR regions has been limited. Additionally, ProteinMPNN-based tools have features such as the occasional re-ordering of antibody chains or CDRH3 position 112 insertions, or the introduction of gaps into IMGT-numbered antibodies, incompatible with antibody structures.

**Figure 1.**
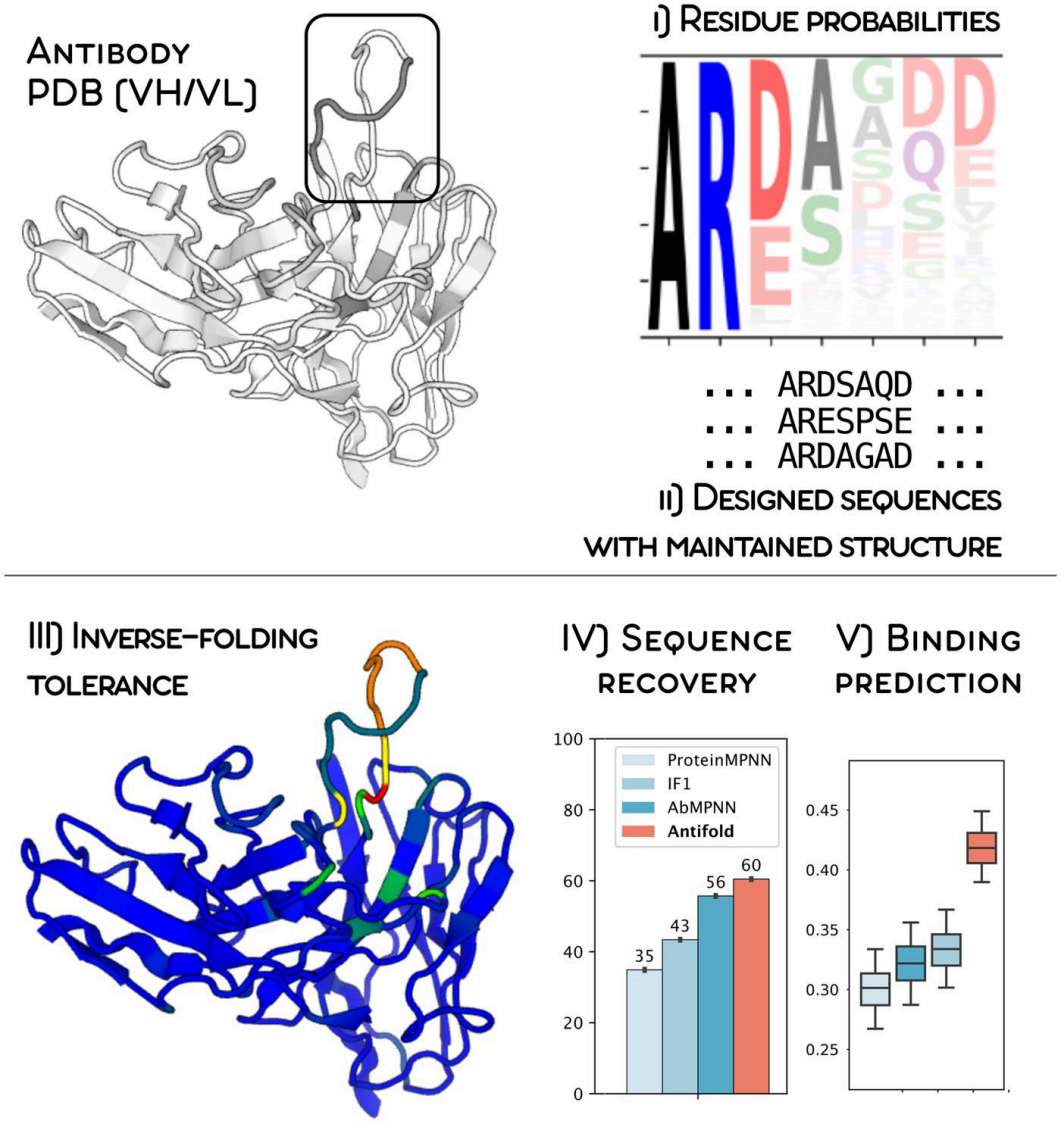
Structure-constrained antibody design with AntiFold. The user inputs an antibody variable domain PDB structure (heavy and light chain) and specifies an IMGT region to design. AntiFold outputs for each residue position in the PDB: (i) residue probabilities, (ii) a number of designed sequences (default 10) for the selected region, predicted to maintain its structural fold, and (iii) structural tolerance to mutations without altering the backbone structure. The diversity of the generated sequences may be controlled with a temperature parameter. AntiFold achieved (iv) state-of-the-art CDRH3 sequence recovery and (v) inverse folding zero-shot binding affinity prediction on an anti-lysozyme antibody dataset (Warszawski *et al*. 2019).

Here, we present AntiFold, an antibody-specific inverse folding model fine-tuned from ESM-IF1 (Hsu *et al.* 2022), which significantly improves upon CDR sequence recovery and zero-shot affinity prediction. AntiFold accepts a solved or predicted antibody variable domain structure as input. For each residue position, the tool outputs the overall tolerance to mutations without altering the backbone structure (perplexity) and individual amino acid probabilities (Fig. 1). Optionally, the user may specify regions to sample new sequences for and a temperature parameter to control sequence diversity. Designed sequences show high structural similarity to the original structure when re-folded. The use of AntiFold in tandem with other property prediction tools could therefore guide antibody optimization campaigns by prioritizing experimental validation to a smaller search space.

## 2 Methods

### 2.1 Data

AntiFold leverages ESM-IF1’s pre-training on >12M protein structures by fine-tuning the model further on solved and predicted antibody structures. To enable a direct comparison with AbMPNN, we trained, validated, and tested our model on the same data: 2074 solved complexes from the Structural Antibody Database (SAbDab) (Dunbar *et al.* 2014,  Schneider *et al.* 2021) and 147,458 structures of sequences from the Observed Antibody Space (OAS) paired database (Kovaltsuk *et al.* 2018, Olsen *et al.* 2022) modeled with ABodyBuilder2 (Abanades *et al.* 2023). Each dataset was split using a 90% concatenated CDR sequence identity cutoff (80/10/10 train/validation/test) (Dreyer *et al.* 2023). We did not directly compare against IgMPNN as the model weights and training data were not available.

### 2.2 Training AntiFold

During fine-tuning, we applied several strategies to improve performance on the validation set, specifically amino acid recovery (AAR) of the heavy chain CDR3 loop (CDRH3), which forms most of the antigen binding site interactions. CDRH3 AAR was improved through the use of a span-masking scheme (36.4%), additional masking of random residues (53.2%), weighting masking toward CDR residues with a 3:1 weight (53.3%), layer-wise learning-rate decay (54.4%), and including predicted structures from OAS (58.4%) (Supplementary Tables S1–S3). These augmentations were all included in the final AntiFold model. Training AntiFold without the ESM-IF1 pre-training yielded a CDRH3 AAR of 31.5% on the validation set (Supplementary Table S4), highlighting the value of ESM-IF1’s large pre-training dataset. For more details on these results and our fine-tuning strategy, see the Supplementary Information.

## 3 Results

### 3.1 Fine-tuning improves amino acid recovery on antibody sequences

AntiFold demonstrated a substantial improvement in AAR on the experimental structure test set as compared to the original ESM-IF1 model (60% vs 43% AAR for CDRH3, *p* < 0.005; Table 1, Supplementary Fig. S2). AntiFold also outperformed AbMPNN for the CDRH3 loop (60% vs 56%), all remaining CDR regions (75%–84% vs 63%–76%), and most FR regions (87%–94% vs 85%–89%, Supplementary Fig. S3). On a subset of the test dataset limited to antibodies with <70% length-matched CDR sequence identity cutoff with any sequence in the training or validation dataset (81%, 178 structures), performance was maintained (ΔAAR < 2.6% for any CDR or FR region). When evaluating trained models, full backbone and sequence context were provided.

**Table 1.** Summary of AntiFold performance on sequence design and binding affinity prediction.^a^

Evaluation	Dataset	Model			
		*ProteinMPNN*	*ESM-IF1*	*AbMPNN*	***AntiFold***
Amino acid recovery, CDRH3 (%, ↑)	AbMPNN test-set	35%	43%	56%	**60%**
Sequence design, sampled CDR loops (RMSD, ↓)	AbMPNN test-set	1.03	1.01	0.98	**0.95**
AbAg binding affinity ($S_{r}$, ↑)	Warszawski, anti-lysozyme DMS	0.30	0.32	0.33	**0.42**
AbAg improved variants (Rank %, ↑)	Hie et al., 7x Ab affinity-maturation	73%	57%	55%	**80%**

a Full backbone and sequence context were provided for all models. For detailed methods and results, see the Supplementary Information. Arrows indicate where higher/lower values are better; the best results are shown in bold. Evaluations from top to bottom: AbMPNN test-set amino acid recovery (Supplementary Fig. S2), backbone RMSD of inverse folding-designed CDR loops (Supplementary Fig. S5), affinity prediction on the Warszawski *et al*. (2019) antibody-antigen deep mutational scan (Supplementary Fig. S8), separation of Hie *et al*. (2023) affinity maturation-improved antibody variants (fold-change > 1.25) (Supplementary Fig. S11); AbAg: antibody-antigen.

We confirmed that AntiFold can be accurately applied to modeled structure inputs by testing it on the test set structures predicted with ABodyBuilder2. AntiFold achieved similar AAR for solved and predicted structures (ΔAAR −0.5%), unlike AbMPNN which performed slightly worse on experimental structures (ΔAAR −2.7%, Supplementary Fig. S2C). AntiFold also maintained performance when applied to an antibody structure predicted with AlphaFold [PDB 7M3N, Supplementary Table S5 (Mirdita *et al.* 2022)].

### 3.2 Predicted sequences show high structural agreement with experimental structures

To assess whether mutations suggested by AntiFold preserve the backbone structure, we sampled and refolded CDR sequences for a set of high-quality structures. We identified 56 antibody structures in the test set that were solved using X-ray crystallography with a resolution below 2.5 Å. Next, we sampled 20 sequences for each antibody using AntiFold, AbMPNN, ESM-IF1, and ProteinMPNN using a residue sampling temperature of 0.20 (for more details, see Supplementary Methods).

We modeled these sequences using ABodyBuilder2, aligned them with the FR backbone of their experimentally solved counterpart, then calculated the root-mean-square deviation (RMSD) over the CDR residues (for more details, see Supplementary Methods). As a baseline, we modeled the true sequences with ABodyBuilder2 (native, Supplementary Fig. S5). AntiFold generated sequences with high structural similarity to the original backbone, with a mean CDR region RMSD of 0.95 Å, versus AbMPNN (0.98 Å), ESM-IF1 (1.01 Å), and ProteinMPNN (1.03 Å). Structure predictions of the native sequences made using ABodyBuilder2 demonstrated a mean CDR region RMSD of 0.63 Å compared to the experimental CDR backbone.

### 3.3 Inverse folding predicts antibody-antigen binding affinity

We assessed the ability of AntiFold and other inverse folding models to predict antibody-antigen binding affinity by applying them to a deep mutational scan of an anti-lysozyme antibody (Warszawski *et al.* 2019). We calculated the log-likelihoods of the 2209 variable domain variants of Fab D44.1 (PDB 1MLC, present in AntiFold’s test set) (Supplementary Fig. S6). As a sequence-only baseline, we included the sequence-based ESM-2 model (650M parameters). AntiFold significantly outperformed the other models with a Spearman’s rank correlation of 0.418, versus ESM-IF1 (0.334), AbMPNN (0.322), ProteinMPNN (0.301), and ESM-2 (0.264) (significance assessed by comparing the 95% confidence interval overlaps). Including the antigen as additional context for the inverse folding models did not result in a statistically significant difference in affinity prediction (Supplementary Figs S9 and S10).

To further explore the ability of AntiFold to guide antibody design by predicting antibody-antigen binding affinity, we applied the model to 124 variants across 7 antibodies generated in protein language model-guided affinity maturation experiments (Hie *et al.* 2023) (see Supplementary Methods).

We first rank-normalized across all possible single amino acid variant scores for each antibody and each model. We then rank-normalized again across this set to assess the rankings of the 124 experimentally measured variants. Using the experimental binding affinity values, variants were separated into lower (fold-change < 0.75), maintained (0.75–1.25), and improved (>1.25) binding affinity groups (Supplementary Fig. S11).

AntiFold achieved significantly improved separation of these groups, scoring the improved variants with a median rank score of 80% (*p* < 0.005), versus ProteinMPNN (73%), ESM-IF1 (57%), and AbMPNN (55%).

### 3.4 Availability and implementation

AntiFold can be accessed as a web server or freely downloaded as a pip-installable package.

- Web server: https://opig.stats.ox.ac.uk/webapps/antifold/
- Code repository: https://github.com/oxpig/AntiFold

## 4 Discussion

AntiFold, fine-tuned from the general protein inverse folding model ESM-IF1, achieved state-of-the-art performance on antibody sequence recovery and refolding of designed sequences. The use of pre-trained weights substantially improved AntiFold’s performance versus training from scratch, while including predicted structures and weighting masking toward individual CDR residues increased sequence recovery further.

AntiFold’s inverse folding probabilities correlate with antibody-antigen binding affinity across multiple independent experiments, likely by identifying mutations that disrupt the structure and antigen binding. These probabilities also synergized with protein language model-suggested variants, supporting that the models learn orthogonal information. Consistent with previous results (Hie *et al.* 2023, Shanker *et al.* 2024), our findings indicate that AntiFold identifies structurally constrained, high-fitness regions of the mutational landscape, predicted to preserve binding.

## Supplementary Material

vbae202_Supplementary_Data

## References

[vbae202-B1] Abanades B , Wong WK, Boyles F et al ImmuneBuilder: deep-learning models for predicting the structures of immune proteins. Commun Biol 2023;6:575–8. 37248282 10.1038/s42003-023-04927-7 PMC10227038

[vbae202-B2] Anand N , Eguchi R, Mathews II et al Protein sequence design with a learned potential. Nat Commun 2022;13:746. 35136054 10.1038/s41467-022-28313-9 PMC8826426

[vbae202-B3] Dauparas J , Anishchenko I, Bennett N et al Robust deep learning-based protein sequence design using proteinmpnn. Science 2022;378:49–56. 36108050 10.1126/science.add2187 PMC9997061

[vbae202-B4] Dreyer FA , Cutting D, Schneider C et al Inverse folding for antibody sequence design using deep learning. In: *The 2023 ICML Workshop on Computational Biology*. 2023.

[vbae202-B5] Dunbar J , Krawczyk K, Leem J et al SAbDab: the structural antibody database. Nucleic Acids Res 2014;42:D1140–6. 24214988 10.1093/nar/gkt1043 PMC3965125

[vbae202-B6] Hie BL , Shanker VR, Xu D et al Efficient evolution of human antibodies from general protein language models. Nat Biotechnol 2023;42:275–83. 37095349 10.1038/s41587-023-01763-2 PMC10869273

[vbae202-B7] Hsu C , Verkuil R, Liu J et al Learning inverse folding from millions of predicted structures. *bioRxiv*, 2022, preprint: not peer reviewed.

[vbae202-B8] Hummer AM , Abanades B, Deane CM. Advances in computational structure-based antibody design. Curr Opin Struct Biol 2022;74:102379. 35490649 10.1016/j.sbi.2022.102379

[vbae202-B9] Ingraham J , Garg V, Barzilay R et al Generative models for graph-based protein design. In: *Advances in Neural Information Processing Systems*, Vol. 32. 2019.

[vbae202-B10] Jing B , Eismann S, Suriana P et al Learning from protein structure with geometric vector perceptrons. In: *International Conference on Learning Representations*. 2021.

[vbae202-B11] Kovaltsuk A , Leem J, Kelm S et al Observed antibody space: a resource for data mining next-generation sequencing of antibody repertoires. J Immunol 2018;201:2502–9. 30217829 10.4049/jimmunol.1800708

[vbae202-B12] Li Z , Yang Y, Faraggi E et al Direct prediction of profiles of sequences compatible with a protein structure by neural networks with fragment-based local and energy-based nonlocal profiles. Proteins 2014;82:2565–73. 24898915 10.1002/prot.24620 PMC4177274

[vbae202-B13] Lu R-M , Hwang Y-C, Liu I-J et al Development of therapeutic antibodies for the treatment of diseases. J Biomed Sci 2020;27:1–30. 31894001 10.1186/s12929-019-0592-z PMC6939334

[vbae202-B14] Makowski EK , Kinnunen PC, Huang J et al Co-optimization of therapeutic antibody affinity and specificity using machine learning models that generalize to novel mutational space. Nat Commun 2022;13:3788. 35778381 10.1038/s41467-022-31457-3 PMC9249733

[vbae202-B15] Makowski EK , Wang T, Zupancic JM et al Optimization of therapeutic antibodies for reduced self-association and non-specific binding via interpretable machine learning. Nat Biomed Eng 2023;8:45–56. 37666923 10.1038/s41551-023-01074-6 PMC10842909

[vbae202-B16] Marks C , Hummer AM, Chin M et al Humanization of antibodies using a machine learning approach on large-scale repertoire data. Bioinformatics 2021;37:4041–7. 34110413 10.1093/bioinformatics/btab434 PMC8760955

[vbae202-B17] Mirdita M , Schütze K, Moriwaki Y et al Colabfold: making protein folding accessible to all. Nat Methods 2022;19:679–82. 35637307 10.1038/s41592-022-01488-1 PMC9184281

[vbae202-B18] O’Connell J , Li Z, Hanson J et al Spin2: predicting sequence profiles from protein structures using deep neural networks. Proteins 2018;86:629–33. 29508448 10.1002/prot.25489

[vbae202-B19] Olsen TH , Boyles F, Deane CM. Observed antibody space: a diverse database of cleaned, annotated, and translated unpaired and paired antibody sequences. Protein Sci 2022;31:141–6. 34655133 10.1002/pro.4205 PMC8740823

[vbae202-B20] Outeiral C , Deane CM. Perfecting antibodies with language models. Nat Biotechnol 2024;42:185–6. 37845572 10.1038/s41587-023-01991-6

[vbae202-B21] Prihoda D , Maamary J, Waight A et al Biophi: a platform for antibody design, humanization, and humanness evaluation. MAbs 2022;14:2020203. 35133949 10.1080/19420862.2021.2020203 PMC8837241

[vbae202-B22] Rabia LA , Desai AA, Jhajj HS et al Understanding and overcoming trade-offs between antibody affinity, specificity, stability and solubility. Biochem Eng J 2018;137:365–74. 30666176 10.1016/j.bej.2018.06.003 PMC6338232

[vbae202-B23] Regep C , Georges G, Shi J et al The h3 loop of antibodies shows unique structural characteristics. Proteins 2017;85:1311–8. 28342222 10.1002/prot.25291 PMC5535007

[vbae202-B24] Schneider C , Raybould MIJ, Deane CM. SAbDab in the age of biotherapeutics: updates including SAbDab-nano, the nanobody structure tracker. Nucleic Acids Res 2021;50:D1368–72. 10.1093/nar/gkab1050 PMC8728266 34986602

[vbae202-B25] Shanehsazzadeh A , Alverio J, Kasun G et al In vitro validated antibody design against multiple therapeutic antigens using generative inverse folding. bioRxiv, 2023, preprint: not peer reviewed.

[vbae202-B26] Shanker VR , Bruun TUJ, Hie BL et al Unsupervised evolution of protein and antibody complexes with a structure-informed language model. Science 2024;385:46–53. 38963838 10.1126/science.adk8946 PMC11616794

[vbae202-B27] Stanfield RL , Wilson IA. Antibody structure. Microbiol Spectr 2014;2. doi: 10.1128/microbiolspec.aid-0012-2013. 26105818

[vbae202-B28] Strokach A , Becerra D, Corbi-Verge C et al Fast and flexible protein design using deep graph neural networks. Cell Syst 2020;11:402–11.e4. 32971019 10.1016/j.cels.2020.08.016

[vbae202-B29] Tennenhouse A , Khmelnitsky LEV, Khalaila R et al Computational optimization of antibody humanness and stability by systematic energy-based ranking. Nat Biomed Eng 2024;8:30–44. 37550425 10.1038/s41551-023-01079-1 PMC10842793

[vbae202-B30] Warszawski S , Borenstein Katz A, Lipsh R et al Optimizing antibody affinity and stability by the automated design of the variable light-heavy chain interfaces. PLoS Comput Biol 2019;15:e1007207. 31442220 10.1371/journal.pcbi.1007207 PMC6728052

