# Supplementary material for: AntiFold: improved structure-based antibody design using inverse folding
Source: Bioinform Adv. 2025 Mar 21;5(1):vbae202. doi: 10.1093/bioadv/vbae202 (PMC11961221; doi:10.1093/bioadv/vbae202)
Supplement: vbae202_Supplementary_Data [file vbae202_supplementary_data.pdf]

Supplement to

AntiFold: Improved structure-based antibody design using inverse folding

Magnus Haraldson Høie[1,†], Alissa M. Hummer[2,†], Tobias H. Olsen[2], Broncio Aguilar-Sanjuan[2], Morten Nielsen[1] and Charlotte M. Deane[2,*]

[1]Department of Health Technology, Section for Bioinformatics, Technical University of Denmark, Denmark and [2]Department of Statistics, University of Oxford, United Kingdom

[†]These authors contributed equally to this work.

[*]Corresponding author deane@stats.ox.ac.uk

Supplementary Information

Supplementary Methods

Experimental antibody structures from SAbDab

The AbMPNN dataset contains 2,074 structures of antibodies in complex with a protein antigen, after filtering for redundancy and experimental resolution <5 Å (Dreyer et al., 2023). We obtained structures of the corresponding variable fragment domains (Fig. S1), numbered with the IMGT antibody numbering scheme (Lefranc et al., 2003), from SAbDab (Dunbar et al., 2014; Schneider et al., 2021). We modeled structures of the validation and test set using ABodyBuilder2 (Abanades et al., 2023) to evaluate AntiFold performance on predicted structures. One and three structures were removed from the validation and test datasets, respectively, as these could not be modeled with ABodyBuilder2 (Abanades et al., 2023).

Predicted antibody structures from ABodyBuilder2

The structures of 148,832 paired antibody sequences from OAS (Kovaltsuk et al., 2018; Olsen et al., 2022) modeled using ABodyBuilder2 were released as part of ImmuneBuilder (Abanades et al., 2023). Filtering out structures with identical concatenated CDRs, as in AbMPNN (Dreyer et al., 2023), resulted in a dataset of 147,458 structures.

Fine-tuning strategy

We trained AntiFold by fine-tuning the ESM-IF1 inverse folding architecture (Hsu et al., 2022) (Fig. S1) on antibody structures. The inverse folding problem can be formalized as learning the conditional probability distribution, $p(Y|X)$, of the protein sequence, Y, consisting of amino acids $(y_1, \ldots, y_i, \ldots, y_n)$, given the structure, X, with spatial coordinates of the backbone atoms (N, C_α and C) $(x_1, \ldots, x_i, \ldots, x_{3n})$ (Equation (1)) (Hsu et al., 2022).

$$p(Y|X) = \prod_{i=1}^{n} p(y_i|y_{i-1}, \ldots, y_1; X) \quad (1)$$

The ESM-IF1 architecture consists of 4 Graph Neural Network Geometric Vector Perceptron (GVP-GNN) layers (Jing et al., 2021), 8 generic Transformer (Vaswani et al., 2023) encoder layers and 8 decoder layers (Hsu et al., 2022). The architecture is invariant to rotation and translation of the input coordinates.

The ESM-IF1 model is trained only on single chain structures. In order to represent complexes of antibody heavy and light chains, we concatenated the backbone coordinates of the light chain to the end of the heavy chain, with a 10 position padding of "gap" tokens, represented as missing coordinates in the input structure.

Fine-tuning parameter evaluation

We evaluated the effect of the parameters described below on model performance, as applied to the validation dataset.

Layer-wise learning rate decay

We decayed the learning rate for each previous layer in the ESM-IF1 architecture by an alpha factor:

$$LR_i = LR \times \alpha^i \quad (2)$$

where i ranges from zero to the number of layers in the model (20), and alpha is set to 0.85.

Masking

We masked portions of the input antibody structure for model training and calculated loss over model predictions for the masked positions. The coordinates of masked positions were hidden for input to the model.

We evaluated three different masking schemes:

- Shotgun masking: individual positions for masking were randomly selected
- Span masking: spans (consecutive stretches of positions) were masked by randomly selecting starting positions and sampling the span length from a geometric distribution where p = 0.05, with a maximum span length of 30 positions, as in (Hsu et al., 2022)
- Shotgun plus span masking: 7.5% of the structure was first masked using span masking and a further 7.5% was subsequently masked using the shotgun approach

Antibody sequence/structure can be separated into FR and CDR regions (Fig. S1A), with the former being more conserved and typically easier to predict. As our model loss is calculated over masked positions, we explored whether performance could be improved by biasing the selection of masked positions towards CDR residues (IMGT-weighted masking). There are more than 2.5 times as many FR as CDR positions in the sequence. For shotgun masking, we implemented a 3:1 weighting for the selection of CDR versus FR positions. For span masking, we biased selection to be low (weight = 1) for most FR positions, high (weight = 3) for most CDR positions, and medium (weight = 2) for FR positions immediately preceding CDRs as well as CDR positions immediately preceding FRs.

Gaussian noise

For predicted structures, we added noise to the backbone (N, C_α and C) 3D-coordinates, sampled from a Gaussian distribution with a scale of 0.1 Å, following the approach taken in ESM-IF1 (Hsu et al., 2022).

Early stopping

Model training was stopped when validation loss did not decrease after 10 epochs. The model with the lowest validation loss was carried forward.

Model parameters

The final AntiFold hyperparameters used were a learning rate of 1e-04, the AdamW PyTorch optimizer, a weight-decay factor of 0.997 per ESM-IF1 defined layer, a batch size of 80 and a backbone masking rate of 15%, as specified above.

Model performance evaluation

Amino acid recovery (AAR) is calculated as the percent of positions for which the top predicted amino acid is identical to the observed amino acid in the PDB.

$$\text{AAR} = \left(\frac{\sum_{i=1}^{N} \mathbf{1}[\text{argmax}(probs(i)) = \text{obs. AA}(i)]}{N} \right) \times 100\% \quad (3)$$

where N is the total number of positions, and argmax(probabilities(i)) is a function that selects the amino acid with the highest probability at position i.

When calculating amino acid recovery, the models were provided full backbone input and conditioned on full sequence input (fixed backbone design, no masking) unless otherwise specified.

Model output probabilities were given by:

$$logits = raw\ model\ outputs \tag{4}$$

$$probabilities(i) = \frac{e^{\text{logits}(i)}}{\sum_{j=1}^{20} e^{\text{logits}(j)}} \tag{5}$$

Perplexity for each position was given by:

$$perplexities = 2^{-\sum_{i=1}^{20} probabilities(i) \times log_2(probabilities(i))} \tag{6}$$

During sequence sampling, we sampled residues for each position in the CDRs proportional to their probability, using a temperature of 0.20. Here, a value of 1.00 represents sampling directly according to the model output distribution, while a value of ∼0.00 acts like an argmax function, ensuring only the top probability amino acid for each position is selected. We used the same method as ProteinMPNN (Dauparas et al., 2022) of applying temperature directly to the logits before converting to probabilities:

$$scaled\ logits = \frac{logits}{t} \tag{7}$$

ProteinMPNN (Dauparas et al., 2022) and AbMPNN (Dreyer et al., 2023) were run with default settings and the flags –conditional_probs_only, –sampling_temp 0.20, –num_seq_per_target 20 and –seed 37. Sampled sequences were then predicted with ABodyBuilder2 (Abanades et al., 2023) at default settings. We corrected for ProteinMPNN reordered chains, reversal of IMGT position 112 insertions and invalid gaps.

We calculated RMSD using Pymol's rms_cur method (Schrödinger, LLC, 2015) between the solved and predicted backbone (N, C_α, and C atoms) for each region, after aligning on the framework.

Binding affinity prediction

Inverse folding log-likelihoods were predicted for antibody variants in the Warszawski et al. (2019) deep mutational scan for each of the inverse folding models by inputting the PDB 1MLC, heavy (chain B) and light (chain A) variable domains (IMGT positions 1-128). Providing full backbone and sequence context, we mapped per residue logits to log-likelihoods using the Pytorch function torch.nn.LogSoftmax, and used these to correlate with binding affinity values.

When including the antigen, we provided the antigen chain E (as specified by SAbDab) as an additional input chain to the model.

For ESM-2 (650M), we extracted log-likelihoods for the corresponding antibody sequence. Experimental scores were mapped to a log_2 fold-change and correlated with inverse folding scores directly using scipy.stats.spearmanr (Virtanen et al., 2020).

Structures of antibody variants in the Hie et al. (2023) study were identified by searching the PDB for the extracted antibody sequence and selecting the highest sequence identity match. The X-ray structure with the highest resolution was selected. We identified the following PDBs:

- mAb114: 5fhc_HL
- mAb114_UCA: 5fhc_HL
- MEDI8825: 5jw4_WX
- MEDI8825_UCA: 5jw4_WX
- C143: 7jmp_HL
- REGN10987: 7z0y_HL
- S309: 8df5_HL

Rank normalization

When assessing model ranking of improved amino acid variants, we first rank-normalized all single amino acid variant scores ($N = L \times 20$) for each antibody separately. Next, we selected the 124 experimentally measured variants ($N = 124$) and calculated their ranks using the same formula.

Rank normalization of scores was calculated as

$$\text{Normalized Rank} = \frac{\text{Rank} - 1}{N - 1}$$

Where Rank is the variant's score rank and N is the total number of variants.

Bootstrapping

For bootstrapping, we resampled with replacement 1000 times, with the bootstrapped values used to calculate means and confidence intervals.

Statistical tests

All reported p-values were calculated using the Mann-Whitney one-tailed U test unless otherwise stated.

Supplementary results

Fine-tuning strategy

Fine-tuning from a general protein inverse folding model enabled us to benefit from existing knowledge learned by ESM-IF1, which was trained on millions of structures. We explored the effect of multiple parameters on fine-tuning ESM-IF1 on antibody structures.

When fine-tuning on a new task or domain, there is a risk of "catastrophically forgetting" previously learned knowledge. We therefore applied a strategy of layer-wise learning rate decay, successfully used to fine-tune BERT models (Sun et al., 2019). We evaluated exponentially decaying the learning rate from the last to the first layer, preserving the weights of earlier parts of the model during training (see Fig. S1 and Methods). Layer-wise learning rate decay did not further improve sequence recovery (Supplementary Table A1-3), but we retained it for subsequent training to reduce the risk of overfitting and to maintain generalization towards untested properties.

We also investigated different masking schemes in training. Shotgun masking hides the coordinates of randomly selected single positions, while span masking is applied to a consecutive stretch of positions. As FR and CDR regions in the antibody structure have different levels of variability, we tested biasing the selection of masked positions towards the more variable CDR residues (3x weight, IMGT-weighted masking). In total, 15% of the backbone residues were masked during training (for more details on the masking parameters, see Supplementary Methods). As previously reported (Hsu et al., 2022), we found stronger performance for shotgun than span masking on test structures. However, span masking improved CDR sequence recovery for test cases with masked CDR loops, a realistic design use case (Supplementary Table A1-3). IMGT-weighted masking further improved performance on CDR loops, while only slightly reducing sequence recovery on FR regions (Supplementary Table A1-2).

To improve performance by training on more diverse antibodies, we included a large dataset of 147,458 predicted structures from OAS (Olsen et al., 2022) modelled with ABodyBuilder2 (Abanades et al., 2023), in our fine-tuning strategy. We tested the effects of adding Gaussian noise at a scale of 0.1 Å to the modeled protein backbone, previously found to improve performance (Hsu et al., 2022; Dauparas et al., 2022). We found no substantial effect, but have included it in our final model for robustness towards minor variations in input structures (Supplementary Table A3).

Based on these results, we chose to train the final AntiFold model with IMGT-weighted shotgun and span masking, layer-wise learning rate decay and added Gaussian noise on predicted structures. We note that these augmentations, along with the use of the larger pre-trained ESM-IF1 architecture (142M parameters) instead of ProteinMPNN (1.7M parameters), comprise the main differences with AbMPNN. We split the training of AntiFold into two phases. First we fine-tuned ESM-IF1 on one pass of the training dataset of predicted structures from OAS. Next we fine-tuned the model on the solved training dataset, stopping training when there was no further improvement in validation loss for 10 epochs. This model, termed AntiFold, was used for all subsequent analysis.

Observed variability (perplexity)

We calculated the observed perplexity, a measure reflecting the number of amino acids observed in true antibody sequences aligned by their IMGT positions (without any structural constraints), for each CDRH3 position in the test-set. Here, a value of 1 means only one amino acid is ever observed, while 20 represents all twenty amino acids being observed at equal probability. AntiFold constrains the observed perplexity (∼10-14 observed amino acids, IMGT positions 107-114) down to 4-8 amino acids which are predicted to preserve the backbone structure of the loop, versus ∼6-10 for AbMPNN (Fig. S2C). This, combined with the improved sequence recovery, reflects AntiFold's greater confidence and accuracy in designing structurally-maintained antibody sequences.

Performance by CDRH3 length

AntiFold's recovery performance is lower for antibodies with longer CDRH3 loops (Fig. S7), with a median AAR of 71% for shorter loops (6-9 residues) and 48% for longer loops (16-28 residues).

Germline identity

Sequences sampled with AntiFold have a similar germline identity to the input sequences. For the sequences for which the CDRs were sampled with AntiFold for the refolding analysis (Fig. S5), the V gene germline identity between the structure-respective wild-type and sampled sequence increased by less than 1% on average (0.76% for the heavy V gene and 0.11% for the light V gene).

De-selection of lower binding affinity variants

Applying AntiFold to an anti-lysozyme deep mutational scan (Warszawski et al., 2019), we find that AntiFold effectively separated antibody variants with lower (log_2 fold-change $<$ 0) versus improved (log_2 fold-change $>$ 0) binding affinity (Fig. S8), likely by identifying structurally disruptive mutations which are also likely to disrupt antigen binding. For example, choosing a score threshold where ∼50% of variants are discarded, AntiFold de-selects ∼40% of lower while maintaining >95% of improved binding affinity variants (Fig. S8B, score threshold -11).

AntiFold CDRH3 substitution matrix

We investigated AntiFold's trends in substituting residues in the test set CDRH3 positions by calculating a 20x20 matrix of AntiFold's median inverse folding probabilities. Antifold CDRH3 log probabilities loosely resemble BLOSUM62 substitution trends. (Fig S12).

There are some deviations from symmetry in this matrix which may arise from considerations to maintain the structural fold. For example, a mutation of Trp to Arg is less favourable than Arg to Trp. This likely reflects the more structurally disruptive effect that Trp, a very large residue, is likely to have. Additionally, mutations that affect the CDRH3 loop conformational flexibility (e.g., mutations to Pro, Gly and Cys or mutations from Ala and Gly) may also be less favored.

Supplementary Tables and Figures

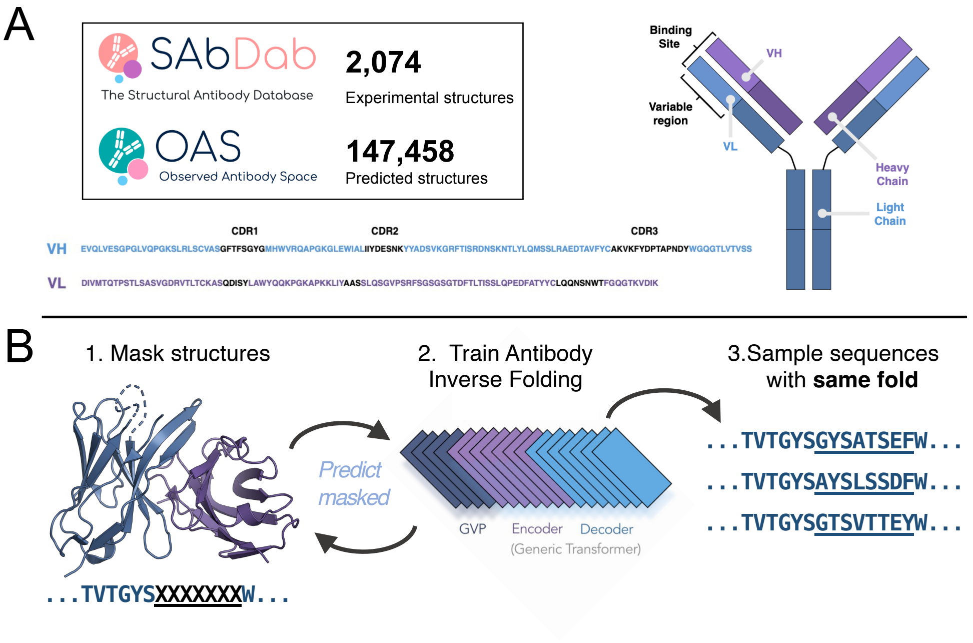

Fig. S1. Overview of the AntiFold training strategy. (A) AntiFold was trained and evaluated on solved antibody structures from SAbDab (Dunbar et al., 2014; Schneider et al., 2021) and structures of antibody sequences from OAS (Kovaltsuk et al., 2018; Olsen et al., 2022) modeled with ABodyBuilder2 (Abanades et al., 2023). Antibodies consist of heavy (blue) and light (purple) chains. Target binding is primarily mediated by complementarity-determining regions (CDRs) in the variable domain. Examples of heavy (VH) and light (VL) variable domain sequences are shown. (B) AntiFold is initialized with weights from ESM-IF1 (Hsu et al., 2022), then fine-tuned on antibody variable domain structures. AntiFold can generate diverse sequences maintaining the fold of the input structure. Figure adapted from (Olsen et al., 2022; Hsu et al., 2022). Structure from PDB 3W2D (Xia et al., 2014).

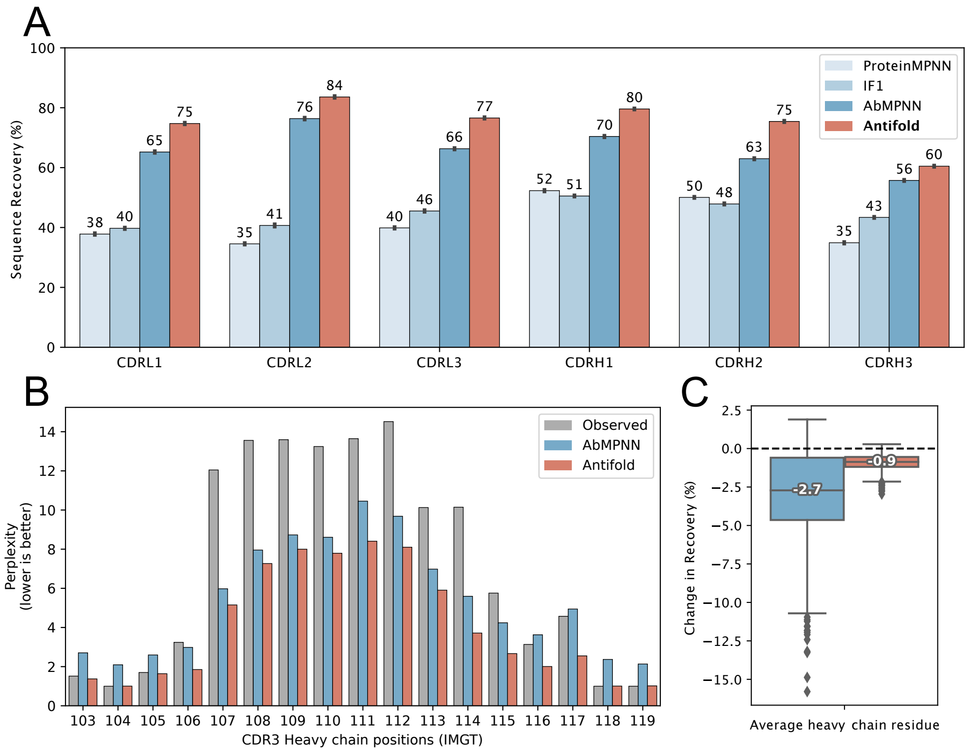

Fig. S2. AntiFold demonstrates improved amino acid recovery and perplexity. (A) Mean amino acid recovery (AAR) across CDRs for antibody heavy and light chains. Error bars indicate 95% confidence intervals after bootstrapping 1000 times with replacement. (B) Perplexity across the CDRH3 loop. Observed perplexity is calculated from all aligned sequences (i.e., with no structural constraints). (C) Percent change in AAR when testing on predicted structures modelled with ABodyBuilder2 (same sequences as the test set of solved structures). For details on these performance evaluations, see Supplementary Methods.

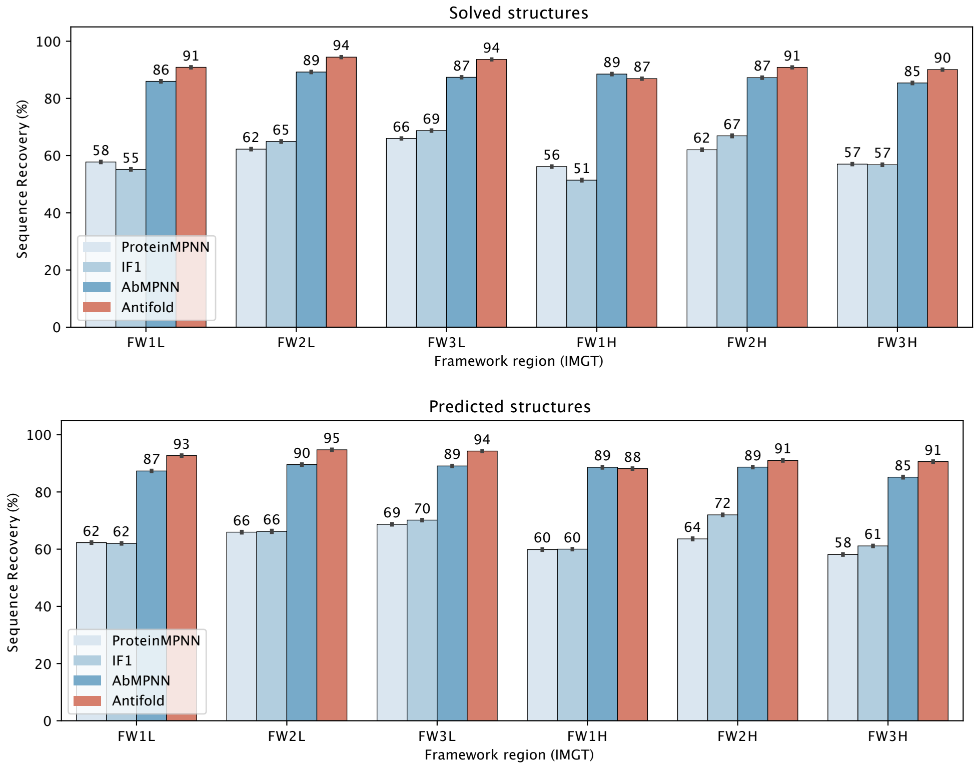

Fig. S3. Framework sequence recovery. Framework amino acid sequence recovery, for solved (top) and predicted (bottom) structures in the test set.

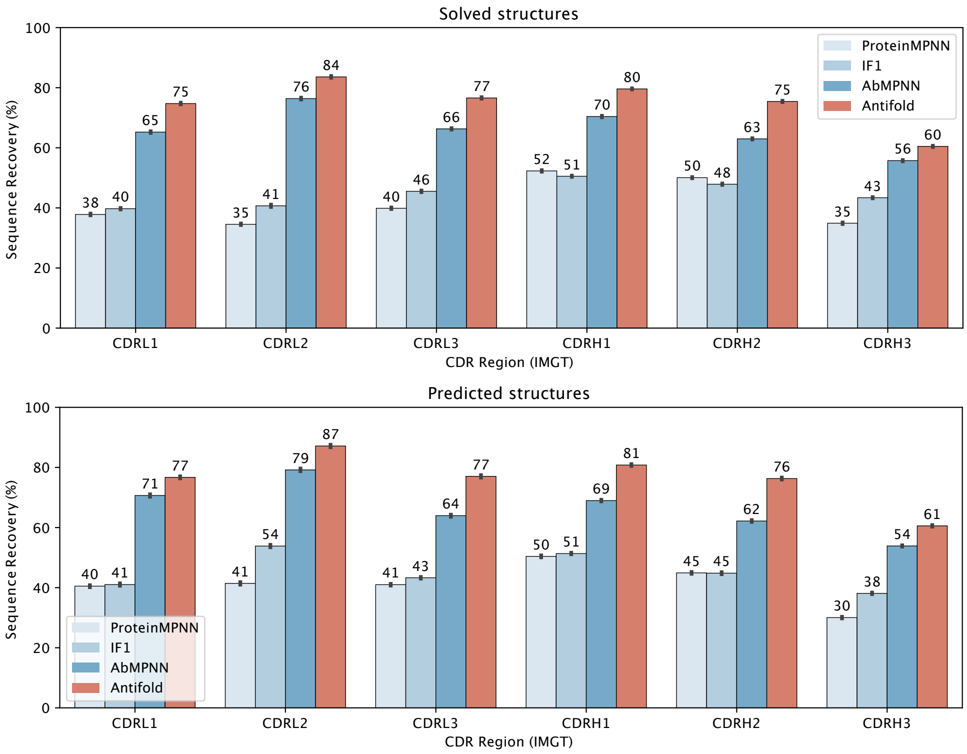

Fig. S4. CDR sequence recovery. Complementarity determining region (CDR) amino acid sequence recovery for solved (top) and predicted (bottom) structures in the test set.

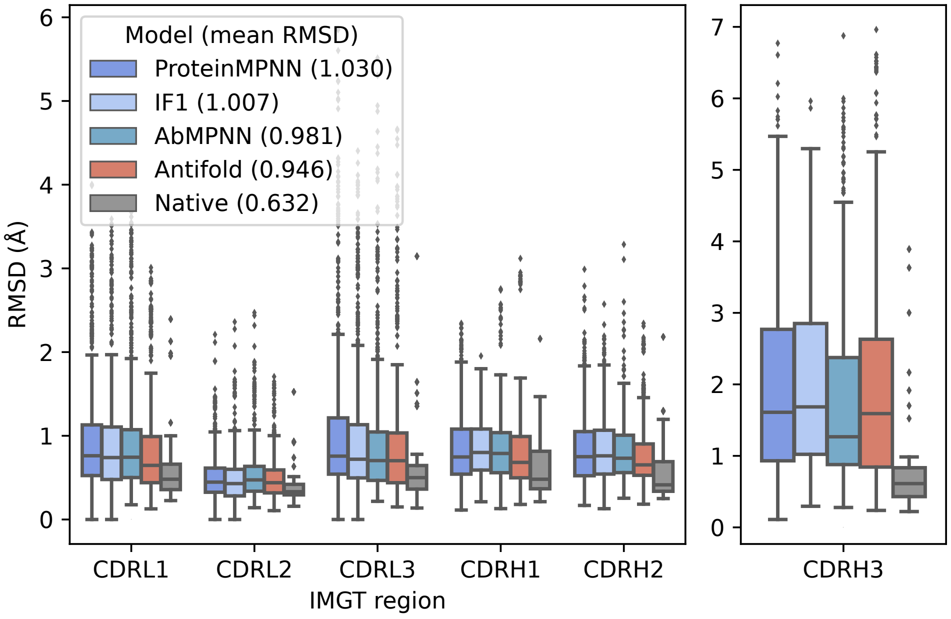

Fig. S5. AntiFold-designed sequences maintain the backbone structure. CDR RMSDs between ABodyBuilder2-predicted and experimental structure backbones, for sequences sampled with ProteinMPNN, ESM-IF1, AntiFold and AbMPNN (temperature 0.20, see Supplementary Methods). CDR RMSDs between the AbodyBuilder2 model of the native sequence and the experimental structures are shown in gray. Mean CDR region RMSD values are shown in parentheses in the legend. We note that the RMSD calculation here only considers the modelling accuracy of the backbone and not side-chain atoms.

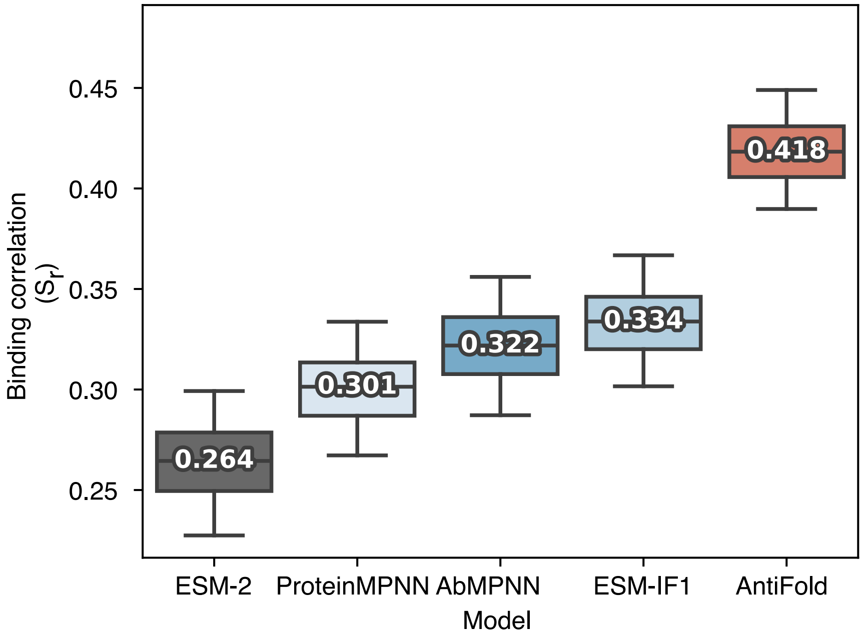

Fig. S6. Inverse folding probabilities correlate with antibody-antigen binding affinity. Spearman's rank correlation between log-likelihood scores of the 2209 variants of the D44.1 anti-lysozyme antibody and the log_2 fold-change in binding affinity Warszawski et al. (2019). Error bars show 95% confidence intervals for the Spearman's rank correlation after bootstrapping 1000 times.

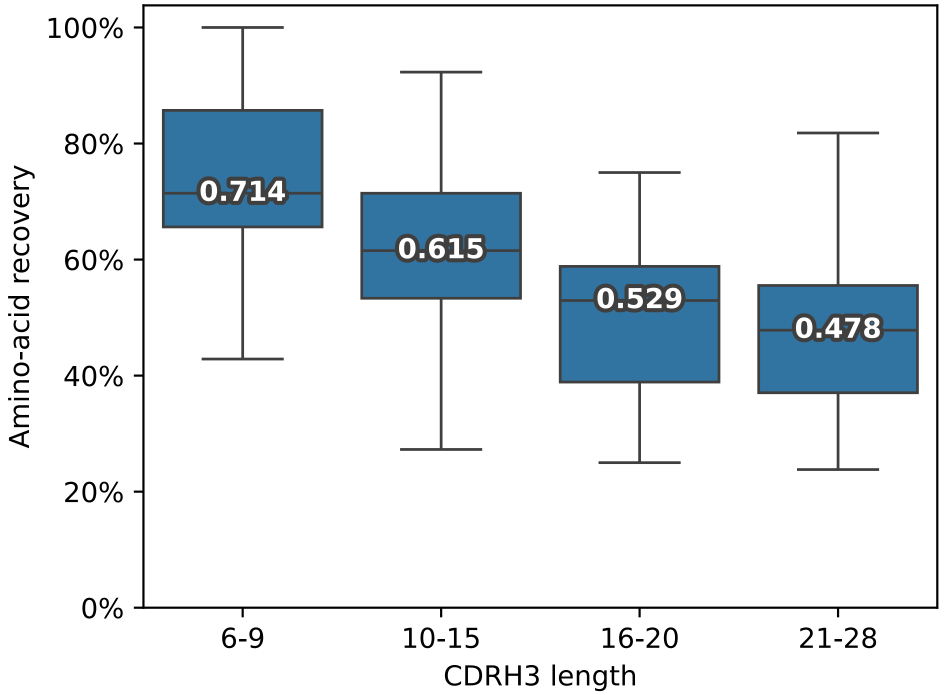

Fig. S7. AntiFold amino acid recovery is higher for shorter CDRH3 loops. Test-set amino acid recovery by CDR3 heavy chain length.

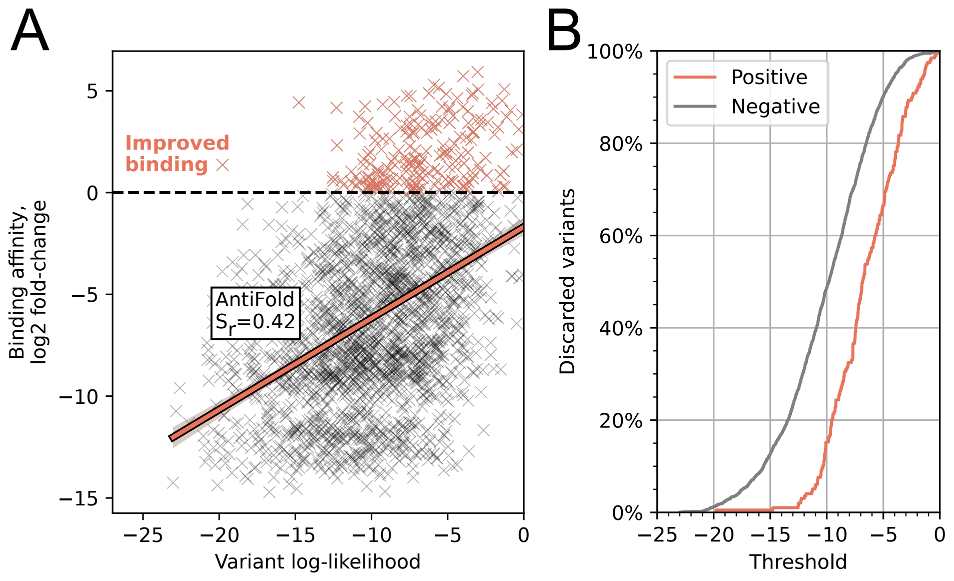

Fig. S8. AntiFold de-selection of lower binding affinity variants (anti-lysozyme deep mutational scan). A) Scatterplot of AntiFold variant log-likelihood versus experimental binding affinity values from Warszawski et al. (2019). Spearman's rank correlation and fitted ordinary least squares model shown. Variants with improved binding affinity (log_2 fold-change > 0) are shown in orange (positives). B) Cumulatively de-selected positive/negative variants at a given score threshold. At a minimum variant log-likelihood threshold of -11, ca 40% of negatives and 5% of positives were de-selected.

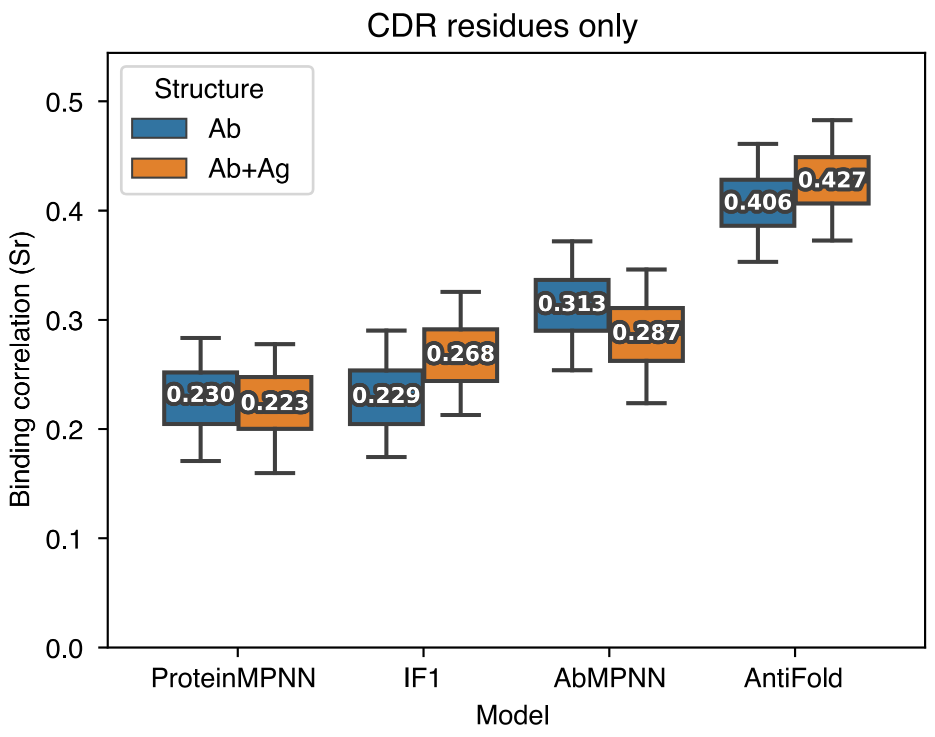

Fig. S9. AntiFold binding affinity prediction with antigen information. Spearman's rank correlation between inverse folding model scores and experimental affinity values in the Warszawski et al. (2019) deep mutational scan, excluding and including the antigen chain. Values bootstrapped 1000 times.

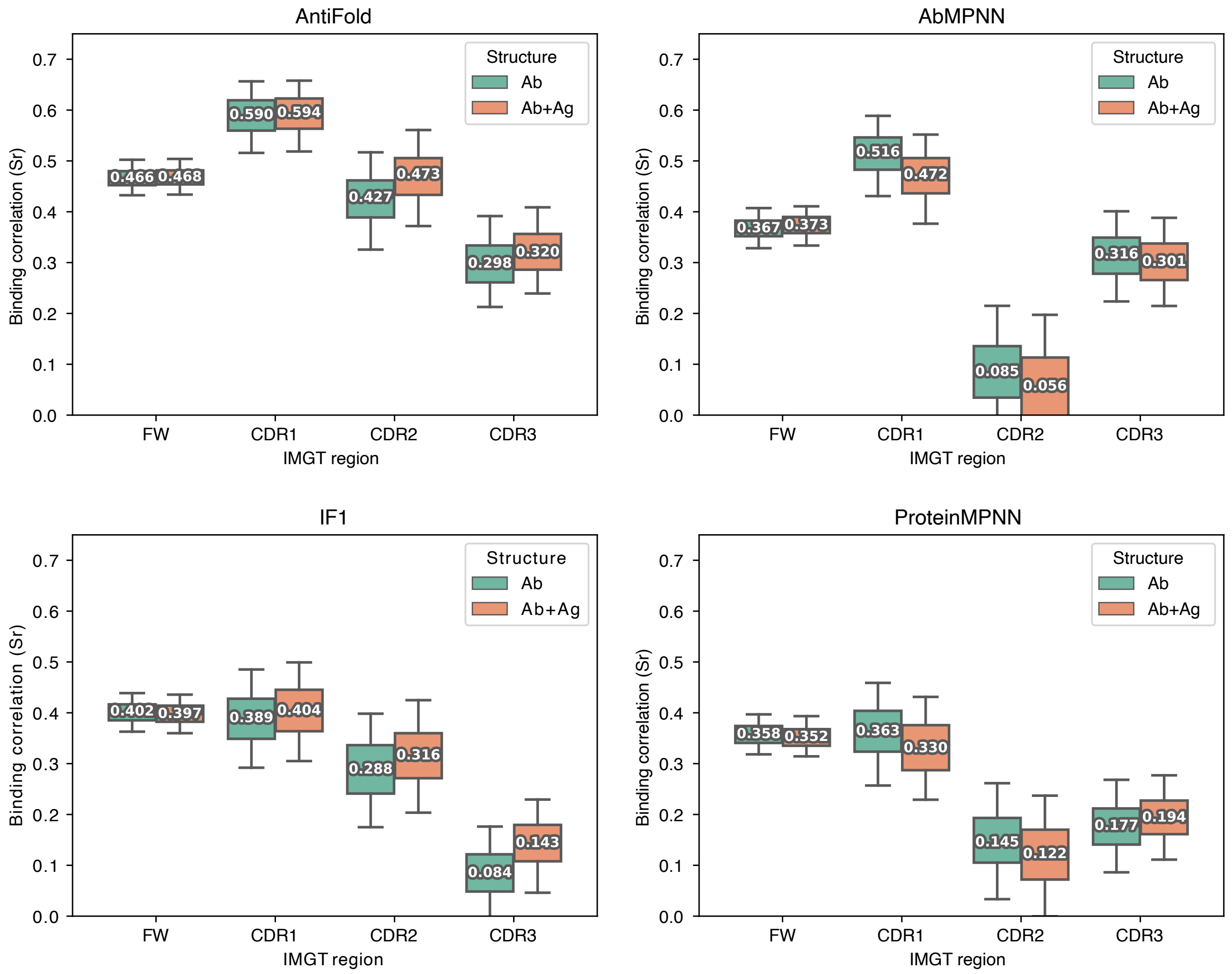

Fig. S10. Model antibody-antigen binding affinity prediction Spearman's rank correlation between inverse folding model scores and experimental affinity values in the Warszawski et al. (2019) deep mutational scan, excluding and including the antigen chain and split by regions. Values bootstrapped 1000 times.

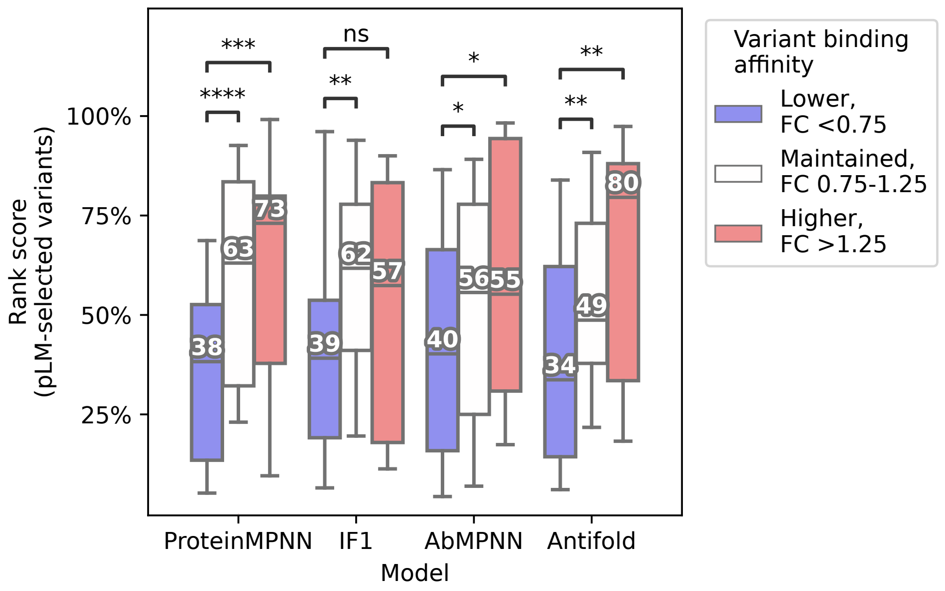

Fig. S11. AntiFold enables de-selection of protein language model-suggested variants. Model rank-scoring performance across 124 variants of 7 antibodies from protein language model-guided affinity maturation experiments by Hie et al. (2023). Variants are separated into lower (fold-change <0.75), maintained (fold-change 0.75-1.25) and higher (fold-change >1.25) binding affinity groups (see Supplementary Methods for details). Boxplots show the 95% confidence intervals of variant scores (rank-normalized across the 124 variants), with median values and Mann-Whitney one-tailed (less) U test shown (** = $p < 0.005$).

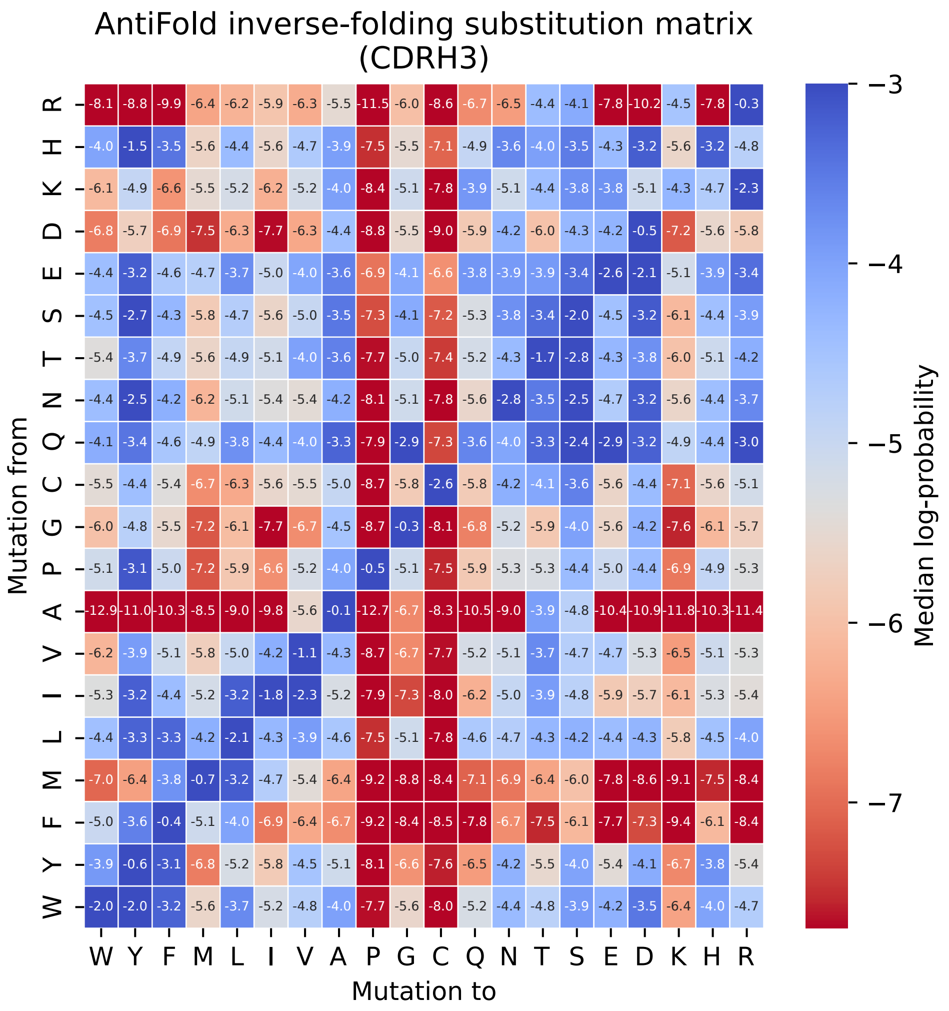

Fig. S12. AntiFold CDRH3 inverse folding probabilities capture physiochemical patterns. Substitution matrix with median residue inverse folding probabilities (log) for mutations in the solved test-set CDR3 heavy chain regions.

Table S1. Fine-tuning parameter evaluation, applied to validation dataset (experimental, "Exp", structures). The training (layer-wise learning rate decay, train masking) and testing (test masking) parameters are indicated. The values in the right side of the table represent amino acid recovery for a particular IMGT-region (FR: framework, CDR: complementarity-determining region). The highest value is shown in bold, the second-highest in italics.

Exp/Pred	Layer Decay	Train Masking	Test Masking	FR Avg.	CDRH1	CDRH2	CDRH3	CDRL1	CDRL2	CDRL3
Exp	–	Shotgun	None	**0.845**	0.695	0.606	0.532	0.597	0.584	*0.609*
Exp	–	Span	None	0.814	0.635	0.506	0.364	0.521	0.516	0.505
Exp	–	Shotgun + Span	None	*0.842*	0.675	0.601	0.525	0.570	0.559	0.582
Exp	–	Shotgun – IMGT-Weighted	None	0.835	**0.708**	**0.640**	*0.543*	**0.613**	**0.628**	**0.626**
Exp	–	Span – IMGT-Weighted	None	0.807	0.636	0.511	0.365	0.535	0.521	0.516
Exp	–	Shotgun + Span – IMGT-Weighted	None	0.837	0.688	0.631	0.533	0.591	0.611	0.601
Exp	✓	Shotgun	None	*0.842*	**0.708**	0.620	*0.543*	0.601	0.567	*0.609*
Exp	✓	Span	None	0.803	0.621	0.500	0.364	0.513	0.492	0.487
Exp	✓	Shotgun + Span	None	0.838	0.684	0.609	0.538	0.587	0.577	0.596
Exp	✓	Shotgun – IMGT-Weighted	None	0.832	**0.708**	*0.636*	0.541	*0.611*	*0.614*	**0.626**
Exp	✓	Span – IMGT-Weighted	None	0.798	0.614	0.498	0.354	0.507	0.502	0.494
Exp	✓	Shotgun + Span – IMGT-Weighted	None	0.833	*0.699*	0.629	**0.544**	0.600	0.598	0.606
Exp	–	Shotgun	CDRs	**0.832**	0.520	0.388	0.310	0.439	0.438	0.437
Exp	–	Span	CDRs	0.811	*0.622*	0.507	0.348	0.521	*0.521*	0.485
Exp	–	Shotgun + Span	CDRs	**0.832**	0.587	0.477	*0.368*	0.506	0.484	0.485
Exp	–	Shotgun – IMGT-Weighted	CDRs	0.827	0.608	0.496	0.343	0.520	**0.545**	*0.499*
Exp	–	Span – IMGT-Weighted	CDRs	0.807	**0.623**	*0.512*	0.354	*0.532*	0.511	**0.509**
Exp	–	Shotgun + Span – IMGT-Weighted	CDRs	*0.828*	0.604	**0.532**	**0.380**	**0.541**	0.511	0.493
Exp	✓	Shotgun	CDRs	*0.828*	0.524	0.386	0.307	0.428	0.446	0.434
Exp	✓	Span	CDRs	0.800	0.599	0.483	0.330	0.494	0.467	0.470
Exp	✓	Shotgun + Span	CDRs	0.825	0.582	0.483	0.348	0.476	0.466	0.465
Exp	✓	Shotgun – IMGT-Weighted	CDRs	0.824	0.580	0.476	0.350	0.478	0.498	0.466
Exp	✓	Span – IMGT-Weighted	CDRs	0.795	0.606	0.508	0.343	0.490	0.479	0.485
Exp	✓	Shotgun + Span – IMGT-Weighted	CDRs	0.822	0.609	0.497	*0.368*	0.509	0.485	0.498

Table S2. Fine-tuning parameter evaluation, applied to validation dataset (predicted, "Pred", structures). The training (layer decay, train masking) and testing (test masking) parameters are indicated. The values in the right side of the table represent amino acid recovery for a particular IMGT-region (FR: framework, CDR: complementarity-determining region). The highest value is shown in bold, the second-highest in italics.

Exp/Pred	Layer Decay	Train Masking	Test Masking	FR Avg.	CDRH1	CDRH2	CDRH3	CDRL1	CDRL2	CDRL3
Pred	–	Shotgun	None	**0.856**	0.703	0.617	0.519	0.600	0.611	0.604
Pred	–	Span	None	0.816	0.639	0.505	0.373	0.531	0.506	0.499
Pred	–	Shotgun + Span	None	0.851	0.697	0.602	0.510	0.580	0.563	0.596
Pred	–	Shotgun – IMGT-Weighted	None	0.850	*0.708*	*0.640*	*0.520*	**0.636**	*0.625*	**0.635**
Pred	–	Span – IMGT-Weighted	None	0.810	0.643	0.506	0.377	0.545	0.519	0.516
Pred	–	Shotgun + Span – IMGT-Weighted	None	0.844	0.701	0.628	0.513	0.589	0.604	0.602
Pred	✓	Shotgun	None	*0.853*	**0.710**	0.626	*0.520*	0.585	0.597	0.603
Pred	✓	Span	None	0.808	0.618	0.487	0.361	0.503	0.464	0.481
Pred	✓	Shotgun + Span	None	0.848	0.693	0.615	0.507	0.587	0.585	0.593
Pred	✓	Shotgun – IMGT-Weighted	None	0.847	0.704	**0.645**	**0.526**	*0.620*	**0.632**	*0.624*
Pred	✓	Span – IMGT-Weighted	None	0.803	0.615	0.509	0.359	0.512	0.499	0.493
Pred	✓	Shotgun + Span – IMGT-Weighted	None	0.842	0.706	0.634	0.518	0.596	0.612	0.605
Pred	–	Shotgun	CDRs	**0.844**	0.535	0.395	0.327	0.438	0.444	0.444
Pred	–	Span	CDRs	0.814	0.618	0.501	0.351	0.517	0.508	0.486
Pred	–	Shotgun + Span	CDRs	0.840	0.603	0.481	0.374	0.517	0.473	0.478
Pred	–	Shotgun – IMGT-Weighted	CDRs	*0.841*	0.622	0.504	0.356	0.522	**0.534**	0.492
Pred	–	Span – IMGT-Weighted	CDRs	0.810	**0.630**	*0.512*	0.356	*0.536*	*0.529*	*0.499*
Pred	–	Shotgun + Span – IMGT-Weighted	CDRs	0.836	*0.627*	**0.536**	**0.394**	**0.537**	0.509	**0.502**
Pred	✓	Shotgun	CDRs	0.840	0.540	0.388	0.319	0.435	0.445	0.426
Pred	✓	Span	CDRs	0.805	0.600	0.488	0.341	0.498	0.481	0.464
Pred	✓	Shotgun + Span	CDRs	0.836	0.600	0.464	0.351	0.494	0.468	0.463
Pred	✓	Shotgun – IMGT-Weighted	CDRs	0.837	0.586	0.476	0.361	0.482	0.498	0.475
Pred	✓	Span – IMGT-Weighted	CDRs	0.800	0.610	0.503	0.344	0.493	0.496	0.487
Pred	✓	Shotgun + Span – IMGT-Weighted	CDRs	0.835	0.612	0.487	*0.377*	0.523	0.471	0.494

Table S3. Final model parameter evaluation, applied to validation dataset (experimental, "Exp", and predicted, "Pred", structures). Each model was trained with IMGT-weighted shotgun plus span masking for 1 epoch on the large predicted OAS structure dataset, followed by training on the experimental SAbDab dataset. The other training parameters (layer-wise learning rate decay and application of Gaussian noise to the predicted OAS structures) are indicated. The values in the right side of the table represent amino acid recovery for a particular IMGT-region (FR: framework, CDR: complementarity-determining region). The highest value is shown in bold, the second-highest in italics.

Exp/Pred	Layer Decay	OAS Gaussian Noise	Test Masking	FR Avg.	CDRH1	CDRH2	CDRH3	CDRL1	CDRL2	CDRL3
Exp	-	-	None	**0.898**	0.731	**0.712**	0.569	**0.723**	*0.736*	0.718
Exp	-	✓	None	**0.898**	*0.735*	0.698	0.566	0.716	0.702	0.713
Exp	✓	-	None	*0.895*	**0.741**	0.700	**0.584**	0.716	**0.741**	*0.725*
Exp	✓	✓	None	0.894	0.727	*0.702*	*0.573*	*0.720*	0.728	**0.727**
Exp	-	-	CDRs	**0.894**	0.680	0.637	*0.432*	*0.677*	*0.689*	**0.661**
Exp	-	✓	CDRs	**0.894**	**0.696**	0.651	**0.434**	**0.692**	0.680	*0.659*
Exp	✓	-	CDRs	0.890	0.675	**0.657**	0.431	0.666	*0.689*	0.658
Exp	✓	✓	CDRs	*0.891*	*0.681*	*0.653*	0.430	0.666	**0.698**	0.655
Pred	-	-	None	**0.909**	**0.753**	*0.716*	*0.561*	0.738	0.731	*0.722*
Pred	-	✓	None	0.905	0.749	0.704	0.558	0.729	0.725	*0.722*
Pred	✓	-	None	*0.907*	*0.750*	**0.730**	**0.572**	**0.746**	**0.737**	**0.730**
Pred	✓	✓	None	0.903	0.744	0.713	0.554	*0.744*	*0.733*	0.718
Pred	-	-	CDRs	**0.904**	*0.706*	0.650	**0.445**	*0.691*	*0.687*	**0.665**
Pred	-	✓	CDRs	0.901	**0.709**	**0.657**	*0.435*	**0.701**	**0.690**	*0.658*
Pred	✓	-	CDRs	*0.903*	0.695	*0.654*	*0.435*	0.675	0.675	0.654
Pred	✓	✓	CDRs	0.898	0.699	0.647	0.433	0.682	0.682	*0.658*

Table S4. The model when developed without ESM-IF1 pretraining (i.e., with weights not initialised from ESM-IF1), with final parameters (layer-wise learning rate decay, IMGT-weighted shotgun plus span masking and application of Gaussian noise to the predicted OAS structures). The values in the right side of the table represent amino acid recovery for a particular IMGT-region (FR: framework, CDR: complementarity-determining region).

**Exp/Pred**	**Test Masking**	**FR Avg.**	**CDR1H**	**CDR2H**	**CDRH3**	**CDR1L**	**CDR2L**	**CDR3L**
Exp	None	0.653	0.548	0.362	0.315	0.338	0.335	0.343
Exp	CDRs	0.651	0.547	0.364	0.314	0.339	0.338	0.345
Pred	None	0.662	0.583	0.387	0.329	0.345	0.315	0.351
Pred	CDRs	0.662	0.583	0.387	0.328	0.347	0.323	0.352

Table S5. AntiFold performance in the case of a solved, ABodyBuilder2- and AlphaFold-predicted antibody structure of median CDRH3 length (∼15 residues). Spearman R correlation with the Solved structure scores is shown.

PDB	Structure	Amino acid recovery (%)	Correlation with Solved (S_r)
7M3N_HL	Solved	81.4%	1.000
	ABodyBuilder2	81.4%	0.941
	AlphaFold	81.4%	0.940
